# Diagnosis and Management of Giant Hepatic Hemangioma: The Usefulness of Contrast-Enhanced Ultrasonography

**DOI:** 10.1155/2013/802180

**Published:** 2013-05-16

**Authors:** Masaki Maruyama, Osamu Isokawa, Koki Hoshiyama, Ayako Hoshiyama, Mari Hoshiyama, Yoshihiro Hoshiyama

**Affiliations:** ^1^Division of Gastroenterology and Hepatology, Department of Internal Medicine, Kashiwazaki Central Hospital, 2-1-25 Ekimae, Niigata, Kashiwazaki 9450055, Japan; ^2^Division of Gastroenterology and Hepatology, Kashiwazaki General Hospital and Medical Center, 2-11-3 Kitahanda, Kashiwazaki, Niigata, 9458535, Japan; ^3^Division of Cardiology, Department of Internal Medicine, Kashiwazaki Central Hospital, 2-1-25 Ekimae, Niigata, Kashiwazaki 9450055, Japan; ^4^Division of Endocrinology and Metabolism, Department of Internal Medicine, Kashiwazaki Central Hospital, 2-1-25 Ekimae, Niigata, Kashiwazaki 9450055, Japan; ^5^Department of Surgery, Kashiwazaki Central Hospital, 2-1-25 Ekimae, Niigata, Kashiwazaki 9450055, Japan

## Abstract

Giant hepatic hemangiomas, though often asymptomatic, may require intervention if rapid growth occurs. The imaging studies including the computed tomography, magnetic resonance imaging, and ultrasonography, and so on are effective for the diagnosis and the management of this tumor; however, due to its size and various patterns of these studies, we need to carefully consider the therapeutic methods. Compared to the cost needed for these modalities, recently developed and approved Perflubutane- (Sonazoid-) based contrast agent enhanced ultrasonography is reasonable and safe. The major advantage is the real-time observation of the vascular structure and function of the Kupffer cells. By this procedure, we can carefully follow the tumor growth or character change in a hemangioma and decide the timing of therapeutic intervention, since abdominal pain, abdominal mass, consumptive coagulopathy, and hemangioma growth are the signs for the therapeutic intervention. We reviewed recent reports about Sonazoid-based enhancement and also showed the representative images collected in our department. This is the first review showing the detailed findings of the giant hemangiomas using Perflubutane (Sonazoid). This review will help the physician in making the decision, and we hope that Sonazoid will gain widespread acceptance in the near future.

## 1. Introduction

Hepatic hemangiomas are the most common benign tumors of the liver and usually do not grow [1]. In cases with giant hepatic hemangiomas, intervention is necessary since clinical symptoms due to rapid growth are present [2, 3]. Detailed follow-up imaging examinations of giant hepatic hemangiomas are essential for relevant medical treatment or surgical intervention. Although computed tomography (CT) or magnetic resonance imaging (MRI) is useful for diagnosing hepatic hemangioma, these modalities are expensive and there are several risks associated with the contrast agents used as well as radiation exposure. There are many recent reports describing contrast-enhanced ultrasonography (US) as being safe, inexpensive, and useful for the diagnosis of focal liver lesions [4, 5]. Perflubutane (Sonazoid) is a new contrast agent for contrast-enhanced US approved only Japan. The only contraindication to its use is an allergy to eggs. Sonazoid contrast-enhanced US is very useful for the diagnosis of hemangioma of the liver, since real-time images can be shown that is unable to be obtained by CT or MRI, and yielding information about Kupffer cell function [6, 7]. However, there have been few reports describing Sonazoid contrast-enhanced US for the diagnosis and followup of “giant hepatic hemangioma.” For the followup of huge hemangiomas to decide the timing of therapeutic intervention, the use of Sonazoid is easy, cost effective, and detailed real-time information can be obtained. For the understanding of the procedure and images, we have shown the representative 2 giant hepatic hemangiomas, with maximum diameters of 17 cm and 6 cm, respectively, observed with Sonazoid contrast-enhanced US. Both tumors showed typical imaging patterns using Sonazoid and its reinjection procedure. We herein review the diagnosis and management of giant hepatic hemangiomas and discuss the usefulness of Sonazoid contrast-enhanced US for the diagnosis, observation, and management of giant hepatic hemangiomas. 

## 2. Diagnosis and Management of Giant Hepatic Hemangioma

Hepatic hemangioma is the most common benign liver tumor and typically remains stable in size [1]. Hepatic hemangioma is diagnosed in most patients using noninvasive studies such as CT, MRI, and US. A hepatic hemangioma shows hypodensity at unenhanced CT. At the enhanced CT, a hepatic hemangioma shows the characteristic pattern, and the tumor existence is represented clearly and shows “nodular peripheral puddling” pattern in early phase and subsequently “fill-in” pattern. In cases where a central scar exists, this portion is not enhanced on delayed phase of dynamic CT. MRI imaging features of hepatic hemangioma are usually low signal intensity and well defined on T1-weighted images and very high intensity on T2-weighted images [8, 9]. Hepatic hemangiomas larger than 4-5 cm demonstrate a nonhomogeneously hypoechoic area on B-mode US. At contrast-enhanced US, hepatic hemangiomas show the characteristic puddling and peripheral-nodular enhancement in the early vascular phase followed by a progressive and centripetal fill-in during the late vascular phase. The real-time images like those of dynamic CT and dynamic MRI can be obtained by contrast-enhanced US. It is the same as that of the enhanced CT; the centripetal fill-in may be incomplete on a central scar [5]. CT angiography can be a particularly valuable preoperative study in patients with large tumors [10]. Hemangiomas in the liver greater than 4-5 cm in diameter are usually defined as giant hepatic hemangiomas, according to several reports. Observation is justified in patients with minimal or no symptoms, even those with giant hepatic hemangiomas. The decision to perform surgical resection or another invasive intervention should not be made based solely on size [11–13]. Indications for surgical interventions are abdominal pain, an abdominal mass, a consumptive coagulopathy (Kasabach-Merritt syndrome), and hemangioma growth. Since the safety of hepatectomy and enucleation with normal liver function is improving, it is essential that radical surgery should be appropriately timed [14–16]. Jiang et al. advocated that liver resection is indicated for giant hepatic hemangiomas causing abdominal discomfort, especially for lesions greater than 20 cm [17]. It is possible for consumptive coagulopathy (Kasabach-Merritt syndrome), spontaneous tumor rupture, and tumor hemorrhage to occur in patients with giant hepatic hemangiomas [18–20]. Therefore, it is important to confirm tumor growth or a change in the character of a hemangioma by performing detailed follow-up imaging examinations. On the other hand, large unresectable hemangiomas have been treated with transcatheter arterial embolization (TAE) [21] and liver transplantation [22, 23]. TAE is reportedly useful for reducing the tumor volume, which could potentially facilitate radical resection [24, 25]. A case of hepatic hemangioma with consumptive coagulopathy was also reported, and TAE appeared to ameliorate the coagulopathy and increase the safety of surgery [26]. As mentioned above, in order to treat giant hepatic hemangioma with optimal timing, close observation is required. 

## 3. The Usefulness of Contrast-Enhanced Ultrasonography 

Sonazoid is the recently developed and approved phase contrast agent for the contrast-enhanced US. It is a powerful modality for the various liver tumors. 

Since the US is safe, inexpensive, has few side effects, and can obtain real-time images comparable to those of dynamic CT, it is useful for achieving meticulous follow-up care of various liver tumors, including giant hepatic hemangioma patients. This agent also provides the function of Kupffer's cells inside of the tumors; therefore, we can determine the possible malignancy. In this section, we show the representative images obtained from 2 tumors in our department.

**Figure 1:**
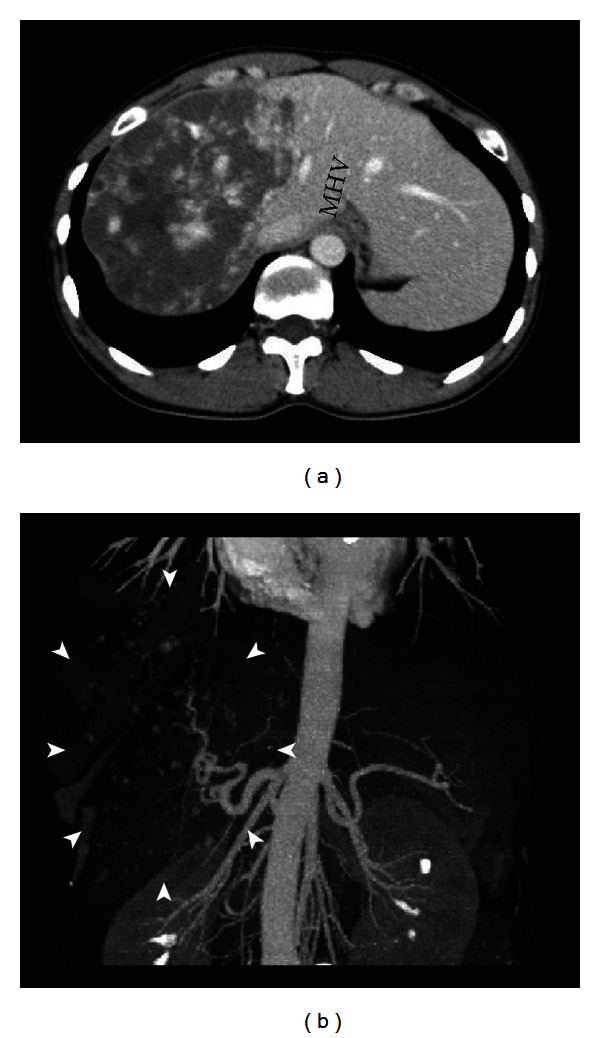
Multidetector computed tomography (MDCT) images. (a) Axial MDCT image shows a huge hypodense mass in the right lobe of the liver measuring about 17 cm, extending to the middle hepatic vein (MHV). (b) Vascular reconstruction image shows “cotton wool-like pooling” (arrowhead).

**Figure 2:**
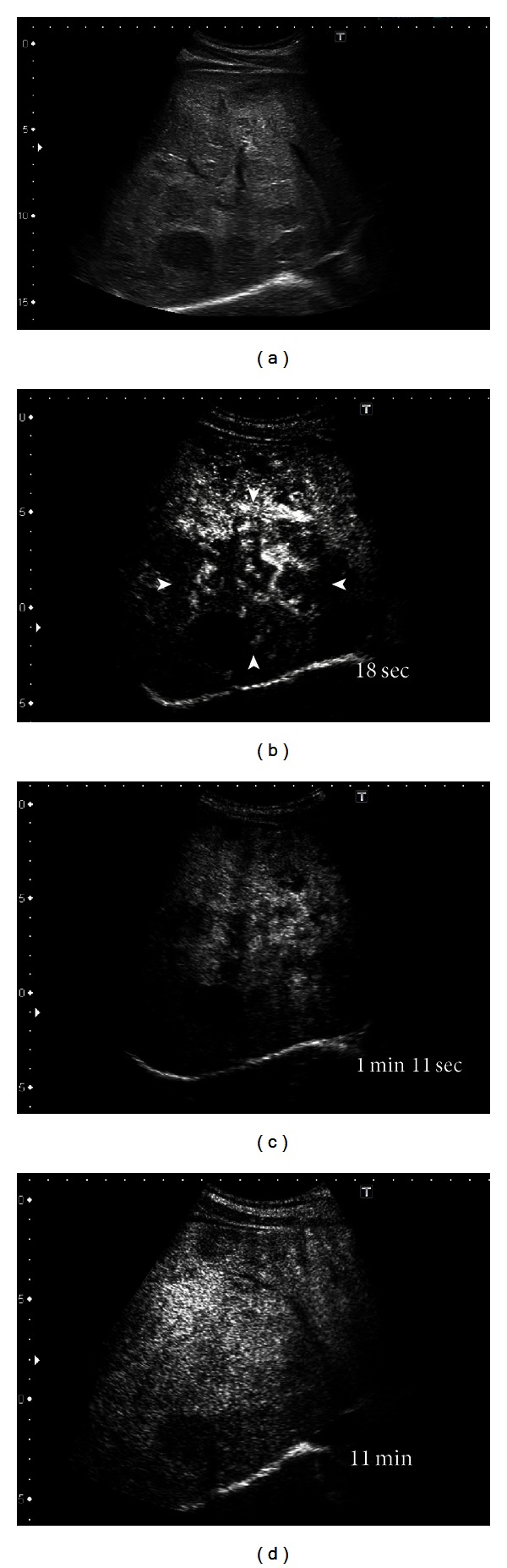
Ultrasonography (US) scan. (a) B-mode US scan shows a huge nonhomogeneously hypoechoic lesion in the right lobe of the liver. (b) Sonazoid contrast-enhanced US in the early vascular phase reveals “cotton wool-like pooling” (arrowhead). (c) Gradual fill-in through the late vascular phase. (d) The fill-in was completed in the Kupffer phase.

**Figure 3:**
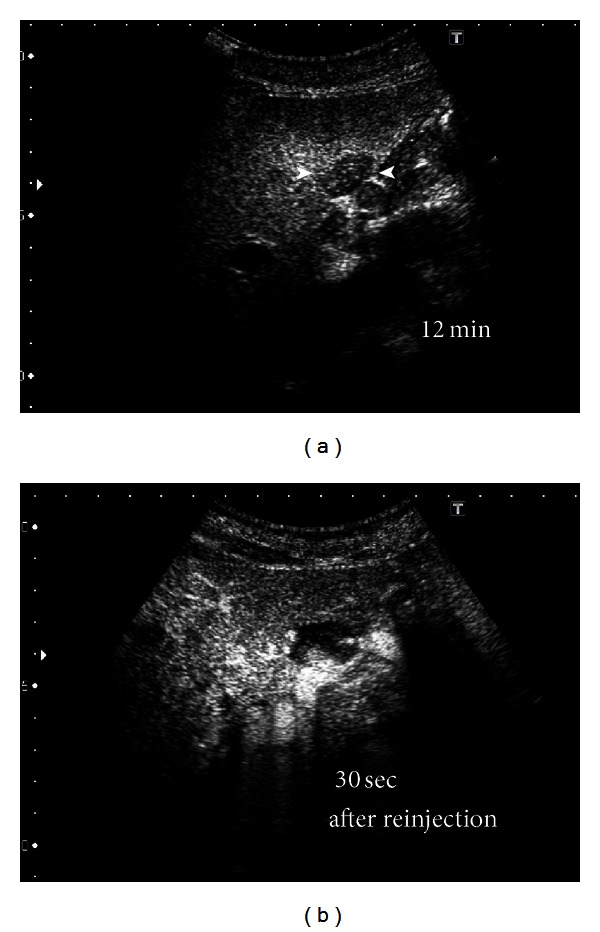
US, employing the “defect reperfusion US imaging” technique with Sonazoid, was performed on another hepatic hemangioma, in the left lobe of the liver, in tumor 1. (a) Preinjection image shows a hypoechoic mass measuring about 2 cm (arrowhead). (b) The lesion shows “peripheral-nodular enhancement” 30 s after reinjection of Sonazoid.

Representative images from tumor 1: multidetector CT (MDCT) revealed a giant hepatic hemangioma, 17 cm in maximum diameter, replacing the right lobe of the liver (Figures 1(a), 1(b), and 2(a)). The hemangioma was near the middle hepatic vein. A small tumor (2 cm) suggestive of hemangioma was also seen in the left hepatic lobe on MDCT. These two tumors showed no changes in either size or character as compared to the MDCT findings obtained 2 years previously. For Sonazoid contrast-enhanced US examination, Sonazoid, injected intravenously as a 0.5 mL bolus followed by 10 mL of normal saline flush using a 22-gauge peripheral intravenous cannula, was used as the US contrast agent. The images showed intratumoral perfusion with “cotton wool-like pooling” in the early vascular phase (Figure 2(b)) and “gradual fill-in” persisting through the late vascular phase (Figure 2(c)). This main tumor showed a slightly inhomogeneous positive uptake in the Kupffer phase (Figure 2(d)). A small tumor, only 2 cm in diameter, suggestive of hemangioma, was also seen in the left lobe in the Kupffer phase (Figure 3(a)). This lesion displayed a slightly negative uptake rather than normal liver parenchymal enhancement. After Sonazoid reinjection according to the US technique reported by Kudo et al., termed as “defect reperfusion ultrasound imaging” [27], we observed slight “peripheral-nodular enhancement” (Figure 3(b)).

**Figure 4:**
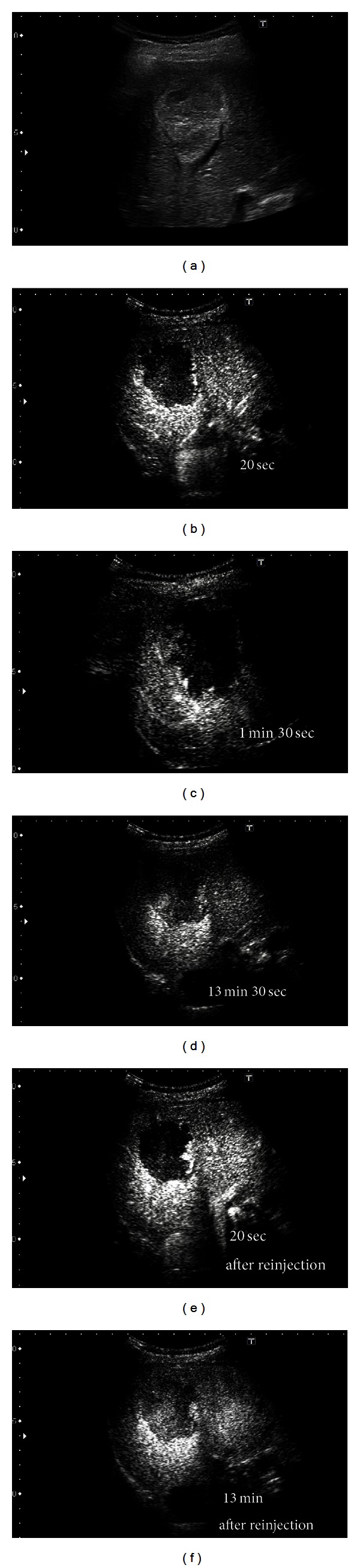
Intranodular dynamic image of the hepatic hemangioma in tumor 2. (a) B-mode US shows a nonhomogeneously hyperechoic lesion approximately 6 cm in size in the right lobe of the liver. (b) Sonazoid contrast-enhanced US in the early vascular phase reveals typical “peripheral globular enhancement” of the lesion. (c) The lesion shows progressive “partial centripetal filling-in” during the late vascular phase. (d) The tumor is visualized as a hypoperfusion image in the Kupffer phase. (e) The same pattern is apparent 20 s after reinjection of Sonazoid: typical “peripheral globular enhancement” of the lesion in the early vascular phase 20 s after the first injection of Sonazoid. (f) Parenchymal perfusion 13 min after reinjection results in a hypoperfusion image similar to that in the Kupffer phase after the first injection of Sonazoid.

Representative images from tumor 2: MDCT showed a huge hypodense mass in the right lobe of the liver measuring approximately 6 cm. In Sonazoid contrast-enhanced US examination (Figure 4(a)), the images showed intratumoral perfusion with “peripheral-nodular enhancement” in the early vascular phase (Figure 4(b)) and “partial centripetal filling” in the late vascular phase (Figure 4(c)). The tumor showed slightly negative uptake in the Kupffer phase with a partial defect (Figure 4(d)). After Sonazoid reinjection in this tumor, according to the “defect reperfusion ultrasound imaging” technique, we observed typical “peripheral-nodular enhancement” and typical Kupffer images 13 min after reinjection (Figures 4(e) and 4(f)).

## 4. Discussion

Contrast-enhanced US with Sonazoid is a useful modality for differentially diagnosing hepatic tumors [6, 7]. Giant hepatic hemangiomas greater than 20 cm in maximum diameter are often reported [24]. In a case with a giant hepatic hemangioma, since the image pick-up range of an ultrasonic probe is narrow, it is difficult to obtain an overall picture of the tumor. However, as Sonazoid contrast-enhanced US is low cost and minimally invasive, it is much easier to scan the hepatic tumor repeatedly. There are few reports describing the observation of giant hepatic hemangiomas with Sonazoid contrast-enhanced US.

We are performing Sonazoid contrast-enhanced US on giant hepatic hemangiomas for the effective followup of the tumors. The giant hepatic hemangioma in representative tumor 1, when observed with Sonazoid contrast-enhanced US (Figure 2(b)), showed intratumoral vessels in the early vascular phase image that has been only obtained by angiography. The findings appeared to represent a general view of both arterial vascularity and portal flow, in practice, despite being in the early vascular phase. In huge hemangiomas, the enhancement pattern is in proportion to the annular form or has a form, in many cases, in which the central portion is a defect due to a central scar. Thus, tumor vessels exist in the periphery of the hepatic hemangioma [28]. Therefore, since an image similar to the projection image of a tumor vessel on angiography will be obtained if the periphery of a giant hepatic hemangioma is scanned on US with tangential adjustment, the typical pattern of a hemangioma on angiography is the so-called “cotton wool-like pooling” which can more easily be represented than “globular pooling.” In our tumor 1, the spatial relationship of a vessel and the corresponding stain portioned was clearly shown. This was considered to be an important finding, greatly facilitating our understanding that the image reflected the blood supply from the hepatic artery to many vascular spaces and thereby clarifying the pathological structure of the hemangioma [8, 9]. If, however, the hemangioma in representative tumor 2, which had a smaller diameter than that in tumor 1, was observed by crossing the image of the tumor at the equatorial plane, rather than calling it “cotton wool-like pooling”, the features known as “puddling” and “centripetal filling” were present (Figures 4(b) and 4(c)). In tumor 1, we recognized the coexistence of a low echoic lesion, which was faint in the Kupffer phase. We examined this low echoic lesion employing the reinjection method known as “defect reperfusion ultrasound imaging” [27] and obtained an image showing “peripheral-nodular enhancement” in the vascular phase (Figures 3(a) and 3(b)). The coexisting lesion was also considered to be a hemangioma, based on these findings. In tumor 2, because there were no other lesions, we performed secondary imaging examinations in the main tumor with Sonazoid contrast-enhanced US according to the “defect reperfusion ultrasound imaging” technique but acquired essentially the same image as the first time (Figures 4(e) and 4(f)). When a coexisting lesion is present, this raises the question of whether blood-flow evaluation is also possible, if Sonazoid contrast-enhanced US is also used to examine another tumor. Moreover, it seems that a second examination is possible, if the first observation of the same lesion was unsuccessful. In this way, if the “reperfusion imaging” is used, Sonazoid contrast-enhanced US may be useful for diagnosis and observation in cases with multiple hemangiomas. Hamaloglu et al. reported that the size average in their series of giant hepatic hemangiomas was 90 mm [29]. Many giant liver hemangiomas with a diameter of 10 cm or less, as on our tumor 2, may also be accurately examined and tested only with Sonazoid contrast-enhanced US and B-mode US with neither CT nor MRI. Furthermore, the Sonazoid contrast agent has a high probability of being useful even in cases with CT contrast media contraindications or relative contraindications such as renal insufficiency, Graves' disease, contrast media allergy, and bronchial asthma. These findings suggest observation of giant hepatic hemangiomas with Sonazoid contrast-enhanced US to be useful, safe, and cost effective.

In conclusion, even giant hepatic hemangiomas usually have nearly the same Sonazoid contrast-enhanced US findings as hepatic hemangiomas with smaller diameters. We observed the “cotton wool-like pooling” finding on Sonazoid contrast-enhanced US of giant hepatic hemangioma to be similar to the image obtained by angiography. This appears to be a very important image for understanding the histological structure of hemangioma. Moreover, Sonazoid contrast-enhanced US is also expected to be useful for the identification and examination of multiple liver hemangiomas. Although the ultrasonic contrast agent Sonazoid is currently a powerful and easy-to-use tool for diagnosis and meticulous follow-up observation of giant hepatic hemangioma, it is, unfortunately, only available in Japan. It is hoped that Sonazoid will gain widespread acceptance in the future.
